# Teams, Rapid Recovery Protocols and Technology to Resume Cardiac Surgery in the COVID-19 Era

**DOI:** 10.21470/1678-9741-2021-0035

**Published:** 2021

**Authors:** Mariana Kabakura do Amaral Lima, Gabrielle Barbosa Borgomoni, Omar Asdrúbal Vilca Mejia

**Affiliations:** 1 Instituto do Coração do Hospital das Clínicas da Faculdade de Medicina da Universidade de São Paulo (INCOR), São Paulo, Brazil.; 2 Hospital Samaritano Paulista, São Paulo, Brazil

**Keywords:** Cardiovascular Surgical Procedures, Quality Improvement, Technology, Patient Safety, Coronavirus Infections

## Abstract

The coronavirus disease 2019 (COVID-19) pandemic brings numerous challenges to the health ecosystem, including the safe resumption of elective cardiac surgery. In the pre-pandemic period, rapid recovery protocols demonstrated, through strategies focused on the multidisciplinary approach, reduction of hospital length of stay, infection rates and, consequently, costs. Even with several studies proving the benefits of these protocols, their acceptance and implementation have been slow. It is believed that the resumption of surgeries in the current context requires the use of rapid recovery protocols combined with the use of a mobile application promoting greater engagement between patients, caregivers and care teams.

**Table:** 

Abbreviations, acronyms & symbols	
COVID-19	= Coronavirus disease 2019
ICU	= Intensive care unit
SARS-CoV-2	= Severe acute respiratory syndrome coronavirus 2
WHO	= World Health Organization

## INTRODUCTION

Severe acute respiratory syndrome coronavirus 2 (SARS-CoV-2), the virus responsible for coronavirus disease 2019 (COVID-19), which has caused the current pandemic ^[1]^, brings with it numerous challenges to the health ecosystem, including the safe resumption of elective surgeries causing a great impact on patients' survival in the cardiac surgery context. Major advances have taken place, such as the creation of the São Paulo Cardiovascular Surgery Registry (REPLICCAR) ^[2]^ and the reduction in mortality reaching international levels ^[3]^. However, in the current scenario it is hazardous to keep performing elective surgeries in a conventional manner.

Although the pathophysiology and evolution to more severe forms and complications caused by COVID-19 are not completely understood, the risk of mortality appears to be higher in male patients, over 60 years old, who have comorbidities such as diabetes mellitus, pulmonary and chronic heart diseases and immunocompromised state ^[4]^. These variables are extremely relevant for the patient undergoing cardiac surgery and, therefore, postponing the surgical procedure may result in catastrophic outcomes and delay in the diagnosis and treatment of patients with coronary disease.

The exact moment for the return of elective surgical procedures is not yet a consensus. This varies according to the number of COVID-19 cases in the region, as well as the infrastructure of health systems and the surgical demand. Urgent cases, which cannot be postponed, will need to rely on efficient and safe systematization. Acquiring a SARS-CoV-2 infection after the surgical procedure can overload the cardiovascular system and be lethal ^[4]^.

The social distancing guidelines for cardiac patients have become a concern, since these individuals clinically decompensate at home, increasing indirect mortality rates by COVID-19 ^[5]^. 

Between 2001 and 2010, the average length of hospital stay for patients undergoing cardiac surgery in developed countries was approximately 10 days, and in underdeveloped countries, more than 12 days ^[6]^. In addition, this time can be extended if there is any complication, directly affecting the time of hospitalization, rehabilitation and costs ^[7]^.

In the pre-pandemic period, rapid recovery protocols showed, through strategies focused on a multidisciplinary and modular approach, a safe reduction in hospital stay ^[8]^, a decrease in the rate of infection acquired in the hospital and, consequently, in costs ^[8-10]^. 

This is a proactive approach to a set of actions that changes the way we manage the care provided to the patient, allowing earlier interventions, optimization of outcomes within the perioperative journey, and discharge based on an expected preparation and guided by patient tolerance ^[11]^. Even patients operated urgently can benefit from these measures, based on the actions implemented after surgery.

Among the several benefits of the strategy, studies show that the average length of hospital stay after surgery was reduced from 6 days to 3 days. Thus, hospital discharge in a shorter period should not be interpreted as an early discharge, but as a consequence of an expected preparation and guided by the patient's tolerance. The results show that patients do not have worse outcomes because they are discharged from hospital in a short time ^[8]^. The reduction of hospital stay becomes one of the most powerful weapons for the resumption of elective surgeries, aiming at lower risk of exposure and contamination by COVID-19, increasing bed turnover and reducing the consumption of hospital resources. Recent studies in bariatric and colorectal surgery also suggest the implementation of rapid recovery protocols for the resumption of elective surgeries. However, some adaptations are necessary, such as the use of treadmills and exercise bikes due to the restriction of the use of common areas within the hospital and the development and improvement of telemedicine both in the preoperative and postoperative care ^[12,13]^.

## COMMENTS

Even with several studies pointing out and proving the benefits of acceleration protocols, their acceptance and implementation have been slow. Several barriers have been encountered, which are related to the patient, the multidisciplinary team and the infrastructure. For a successful implementation, it is necessary for healthcare professionals to modify and adapt the traditional thinking to this ‘new normal’. This care includes team education regarding new evidence-based strategies that demonstrate proactive attitudes such as exhaustive interventions for perioperative glycemic control ^[14]^, smoking cessation, multimodal pain control, adequate nutritional intake, break of prolonged fasting, reincorporation of cardiorespiratory and motor activity as soon as possible, among others.

This line of care focuses on effective communication and the synchronized work of patient-centered teams; this should be the central part for implementing the actions and monitoring the metrics suggested by rapid recovering protocols. In order for this to happen, patients, companions and multiprofessional teams need to be engaged and motivated in the adopted processes and phases. In this scenario, a virtual platform combined with an application could help to align the actions in a synchronized and efficient way, reminding us of the next steps, avoiding failures in adhering to the metrics proposed by the protocol.

The recommendations of the World Health Organization (WHO), Ministry of Health and medical societies were to postpone elective surgery for two reasons: 1) to increase the availability of intensive care unit (ICU) beds and healthcare professionals for patients with severe evolution of COVID-19; and 2) the risk of in-hospital COVID-19 infection and increased patient morbidity and mortality. The social distancing guidelines for cardiac patients have become a concern, since these individuals decompensate at home, increasing indirect mortality rates by COVID-19 ^[5]^. Even with the resumption of elective surgeries, changes in patient care protocols combined with technology are necessary ^[15,16]^.

Restarting elective surgeries with this approach is vital in the context of COVID-19, reducing unnecessary time for patients in the hospital. Therefore, it is essential to implement strategies aimed at assisting patients with indication for highly complex surgeries, such as cardiac surgery, which may be combined with a rehabilitation and remote monitoring program. The growing wave of technological evolution and its relevance in the day-to-day life of the population with mobile devices, especially applications, presents itself as a promising resource in the current global challenge ^[5]^

In underdeveloped and developed countries, scientific production in the field of health promotion through mobile technologies are still rare and dispersed ^[16]^. However, the implementation of health-oriented applications has already shown successful results by generating a positive impact on the educational issue of patients and their families, answering questions in the phases of care, solving communication failures between healthcare professionals and patients, and engaging them in their own care. These resources have potential in the context of the COVID-19 pandemic, since they bring benefits by improving the recovery of patients and restricting the need for return to the hospital ^[17,18]^.

Thus, the use of applications allows the patient's interaction with the multiprofessional team to occur continuously during their perioperative journey and beyond. The patient and their family could have access to the educational content through a friendly and easily accessible interface. In addition, the patient can communicate with the team quickly and efficiently, allowing his/her questions to be answered without having to return to the hospital. Monitoring of the surgical wound through photographs taken by the patient and pain management through pain scales applied by online questionnaires allow early interventions ^[17]^. This also reduces the number of hospital readmissions due to a better patient adherence to the oriented protocols when monitored with the help of technology ^[18]^.

It is believed that performing surgeries inserted in the ‘new normal’ concept requires the use of rapid recovery protocols. It promotes greater engagement among patients, caregivers and care teams who, with the use of a mobile application, persist in the search of continuous improvement of results and, at the same time, in the patients' satisfaction.

**Table:** 

Authors' roles & responsibilities	
MKAL	Substantial contributions to the conception or design of the work; or the acquisition, analysis, or interpretation of data for the work; drafting the work or revising it critically for important intellectual content; agreement to be accountable for all aspects of the work in ensuring that questions related to the accuracy or integrity of any part of the work are appropriately investigated and resolved; final approval of the version to be published
GBB	Substantial contributions to the conception or design of the work; or the acquisition, analysis, or interpretation of data for the work; drafting the work or revising it critically for important intellectual content; agreement to be accountable for all aspects of the work in ensuring that questions related to the accuracy or integrity of any part of the work are appropriately investigated and resolved; final approval of the version to be published
OAVM	Substantial contributions to the conception or design of the work; or the acquisition, analysis, or interpretation of data for the work; drafting the work or revising it critically for important intellectual content; agreement to be accountable for all aspects of the work in ensuring that questions related to the accuracy or integrity of any part of the work are appropriately investigated and resolved; final approval of the version to be published
